# Serum IL-1β, IL-6, IL-8, and TNF-α Levels in Early Diagnosis and Management of Neonatal Sepsis

**DOI:** 10.1155/2007/31397

**Published:** 2008-01-10

**Authors:** A. Nese Citak Kurt, A. Denizmen Aygun, Ahmet Godekmerdan, Abdullah Kurt, Yasar Dogan, Erdal Yilmaz

**Affiliations:** ^1^Department of Pediatrics, Faculty of Medicine, Firat University, Elazig 23119, Turkey; ^2^Department of Immunology, Faculty of Medicine, Firat University, Elazig 23119, Turkey

## Abstract

*Aim*. To determine serum IL-1β, IL-6, IL-8, and TNF-α levels in neonatal sepsis at the time of diagnosis and after therapy, and to show the meaningful on the follow up. *Methods*. This prospective study was performed on newborns who were hospitalized for neonatal sepsis and who were classified as culture-proven sepsis (n=12), as culture-negative sepsis (n=21), and as healthy newborns (n=17). *Results*. At the time of diagnosis, serum IL-1β, IL-6, IL-8, and TNF-α levels of culture-proven sepsis were significantly higher than those of the control groups (P<.05). At the time of diagnosis, IL-1β, IL-6, IL-8, and TNF-α levels of culture-proven sepsis and culture-negative sepsis were significantly higher than levels at the seventh day after antibiotic treatment. *Conclusion*. Serum IL-1β, IL-6, IL-8, and TNF-α are mediators of inflammation and can be used at the diagnosis and at the evaluation of the therapeutic efficiency in neonatal sepsis.

## 1. INTRODUCTION

Bacterial infection in newborns, especially preterm, may rapidly evolve into generalized sepsis. This condition has a gradual and subtle onset, with nonspecific symptoms that may severely compromise the infant’s clinical state if untreated and lead to life-threatening consequences. Neonatal sepsis has a fairly low incidence at birth (1–10/1000 live births) but may affect up to 16% of infants in the neonatal intensive care unit (NICU) with birth weight of 501–1500 gm. The mortality rate is very high: 15–50% of affected infants [1].

Clinical manifestations are nonspecific and laboratory parameters such as white blood cell (WBC) count or C-reactive protein (CRP) are of limited value in identifying infected newborns. As a consequence, appropriate diagnosis and therapy could be delayed, worsening the prognosis of the patient [2–6].

In studies elsewhere [4,7,8], the WBC count showed a low detection sensitivity in neonatal infection. Even the combination of total neutrophil count, immature-to-total neutrophil ratio (I/T), and platelet count failed to reach an appropriate sensitivity and specifity in this pathology. C-reactive protein has been thoroughly studied as a diagnostic tool in neonatal sepsis and also as an indicator of response to therapy [9, 10].

The underdeveloped immune system predisposes preterm newborns to infection, which is a major cause of neonatal morbidity and mortality. Sepsis and endotoxin activate monocytes, macrophages, lymphocytes, fibroblasts, and endothelial cells that produce and secrete IL-1, TNF-α, α-interferon, IL-6, IL-8, and other proinflammatory cytokines. IL-6, stimulated by TNF-α, IL-1, and endotoxin of viral and bacterial infections, acts as a T-cell activation indicator, induces antibody secretion by human B-cells, causes differentiation of cytotoxic T-cells, and also has the ability to inhibit TNF-α production. Moreover, IL-6 is the major stimulant in hepatic protein synthesis, that is, CRP and fibrinogen during acute phase responses. Previous studies have shown that determinations of IL-6 in neonatal blood are of diagnostic value in sepsis. Elevated serum IL-1β, IL-6, IL-8, and TNF-α levels have been found in both the neonatal and adult sepsis. Several studies have evaluated the role of cytokine determinations as early diagnostic markers in neonatal sepsis [11–17].

The aim of this study is to determine serum IL-1β, IL-6, IL-8, and TNF-α levels in neonatal sepsis at the time of diagnosis and after therapy and to show the meaningful on the follow up.

## 2. MATERIAL AND METHODS

This prospective study was performed on newborns who were hospitalized for neonatal sepsis at NICU. Inclusion criteria were positive clinical signs of sepsis and/or history of factors associated with increased risk for infection and parental informed consent. Exclusion criteria were congenital malformations, congenital infections associated with the TORCH complex, and refusal of parental consent. Clinical signs of sepsis were defined as the presence of three or more of the following categories of clinical signs: apnea, tachypnea (>60/min), nasalflaring, retraction, cyanosis, respiratory distress-bradycardia (<100/min), tachycardia (>180/min), hypotonia, seizures-poor skin colour, capillary refilling time longer than two seconds, irritability, and lethargy. Historical factors associated with increased risk for infection included premature rupture of the membranes (in term infants >18 hours), maternal fever during labour, intraamniotic infection, and chorioamnionitis. Two or more abnormal values of the sepsis screen (as white blood cell count <4000 or >10.000 mm^3^, immature-to-total neutrophil ratio higher than 0.2 and CRP positivity) were considered as supportive for diagnosis of infection [18]. Newborns were classified as culture-proven sepsis (positive blood culture), as culture-negative sepsis (negative blood culture, but clinical signs of sepsis with positive sepsis screen and/or a history of risk factors, and antibiotic treatment longer than 7 days), and as control groups (healthy, noninfectious newborns). Blood samples were obtained at time of diagnosis and seventh day after antibiotic treatment, their serum extracted and IL-1β, IL-6, IL-8, and TNF-α were determined.

Blood analysis was done in Firat Medical Center Immunology Laboratory (Elazig, Turkey). The local ethics commission approved the study.

CRP was determined by the Behring Nephelometer 100 Analyzer BN II (NY, USA). Detection limit was 3 mg/L and a serum value of >8 mg/L was defined as abnormally elevated. The serum samples of the study and control groups were studied by ELISA for IL-1β, IL-6, IL-8, and TNF-α levels with human cytokine kits (Biosource, Calif, USA). Detection limit for serum IL-1β level was 1 pg/mL, measure range was 0–250 pg/mL. Detection limit for serum IL-6 level was 2 pg/mL, measure range was 0–500 pg/mL. Detection limit for serum IL-8 level was 5 pg/mL, measure range was 0–1000 pg/mL. Detection limit for serum TNF-α level was 1.7 pg/mL, measure range was 0–1000 pg/mL The collected blood samples were centrifuged at 2500 g for 10 minutes at 4°C. The serum layer was separated and frozen at −80°C for cytokine analysis, which was performed in less than 2 weeks. Freezing/thawing cycles were avoided.

Statistical analyses were performed using the SPSS 11.0 programs for Windowns XP. The results were done as mean ± standard deviation. Kruskal Wallis and post hoc test Scheffe procedures were used; the difference in three groups and P<.05 was considered to be significant. Wilcoxon test was used for the interpretation of the difference between at time of diagnosis and after therapy.

## 3. RESULTS

In total, 50 newborns were included in the study: 12 culture-proven sepsis, 21 culture-negative sepsis, and 17 control. Table 1 shows the characteristics of the study group.

At time of diagnosis, serum IL-1β, IL-6, IL-8, and TNF-α levels of culture-proven sepsis were significantly higher than those of the control groups (P<.05); but only serum IL-8 levels of culture-proven sepsis was significantly higher than culture-negative sepsis (P<.05).

At time of diagnosis, serum IL-1β, IL-6, IL-8, and TNF-α levels of culture-proven sepsis and culture-negative sepsis were significantly higher than levels at seventh day after antibiotic treatment (P<.05).

Serum IL-1β, IL-6, IL-8, and TNF-α levels were showed in Table 2.

## 4. DISCUSSION

Bacterial infection continues to be the major cause of morbidity and mortality in the newborn. Because the prognosis for sepsis largely depends on early identification and treatment, these newborns are subjected to extensive diagnostic evaluation and empirical systemic antibiotic treatment, pending laboratory results. The definitive diagnosis of sepsis is made by a positive blood culture, which requires a minimum of 48–72 hours, yields a positive result in only 30–70% of cases, and may not always be available in peripheral health centers. Several studies have examined the laboratory findings associated with sepsis [19–21]. There is, however, a lack of consensus on the essential tests that would identify newborns with acute infection. Fowlie et al. [22] conducted a systematic review to determine the value of diagnostic tests for bacterial infection in early life (from birth to 90 days old) and reported that the accuracy of tests varies enormously and the tests are of limited value in the diagnosis of infection.

Hematological parameters have been evaluated in previous studies. Da Silva et al. [23] found significant heterogeneity across these studies. The possible sources of heterogeneity were population, age, whether the subjects were at term or preterm, methodological quality, different leukocyte indices, different cutoff values, and interpretation of test results by different laboratory observers.

In the past few decades, it has been observed that several mediators of inflammation tend to become elevated during sepsis. The concentrations of some proinflammatory cytokines, especially TNF-α, IL-6, and IL-8, in systemic circulation were reported to increase in severe infections and septic shock [24]. Martin et al. [17] showed that serum IL-6, IL-8, and TNF-α levels were all higher in septic than in nonseptic newborns.

The study of IL-1β and TNF-α, cytokines that are synthesized at the beginning of the inflammatory cascade has rendered differing results. In our study, the serum TNF-α and IL-1β levels were significantly increased in newborns with sepsis. Results of different published studies relation to these cytokines are contradictory. Concerning IL-1β results, similar to ours, they have been reported by some researchers while not by others [25–28]. Publishing data regarding TNF-α is also divergent. Some studies found the diagnostic utility of this cytokine [25, 26] while others demonstrated similar or even lower levels in infected newborns compared to healthy newborns [29, 30]. Discrepancies in results among different studies could be explained by the variations in laboratory methods in performing the analysis, the time of the sample collection, or the control population selected [28].

Interleukin-6 has been reported as an early indicator of neonatal sepsis because of its rapid increase after endotoxin challenge. IL-6 is secreted by monocytes and macrophages in response to bacteremia [31]. Previous studies have shown IL-6 to be a useful marker of early infection in the newborn [31–34]. Kantar et al. [31] showed that septic preterm newborns had significantly elevated IL-6 levels at the onset of sepsis as compared to the recovery period and the controls. In our study, it was observed that IL-6 levels of newborn with culture-proven sepsis and culture-negative sepsis were significantly higher than controls (P<.05).

Interleukin-8 is a cytokine that has a role in the release, activation, and chemotaxis of neutrophils. Serum IL-8 level has been reported to increase in neonatal sepsis and have a sensitivity of about 80–90% and a specifity of about 76–100% [35, 36]. In this study, it was detected that IL-8 levels of newborns with culture-proven sepsis were significantly higher than culture-negative sepsis and controls (P<.05). Kocabas et al. [37] and Martin et al. [17] found that IL-8 were higher in septic than in nonseptic newborns.

Another characteristic of the markers that are used in the diagnosis of neonatal sepsis is that it gives information about the prognosis of the disease and helps in coming to a decision as to whether to stop or continue antibiotic treatment. In this study, it is found that IL-1β, IL-6, IL-8, and TNF-α levels were statistically decreased in newborns after seven-day therapy than in newborns at the time of diagnosis (P<.05). Similar results have been obtained in many studies [37–39].

## 5. CONCLUSION

Serum levels of IL-1β, IL-6, IL-8, and TNF-α are mediators of inflammation and can be used at the diagnosis and at the evaluation of the therapeutic efficiency in neonatal sepsis.

## Figures and Tables

**Table 1:** Characteristics of the study groups.

Character	Culture-proven sepsis (n=12)	Culture-negative sepsis (n=21)	Controls (n=17)
Gender M/F	8/4	9/13	8/9
Weight(g)	2033±938	2111±975	2294±761
Gestational age (weeks)	34.2±4.0	34.0±3.3	35.3±2.6

**Table 2:** Serum IL-1β, IL-6, IL-8, and TNF-α levels of the study groups.

Serum cytokine levels		Culture-proven sepsis^a^ (n=12)	Culture-negative sepsis^b^ (n=21)	Controls^c^ (n=17)	*P value <.05
IL-1β (pg/mL)	At time of diagnosis^1^	41.20±13.57	33.30±8.62	10.35±2.65	a-c, b-c
	7th day^2^	10.87±4.49	9.47±3.53		
IL-6 (pg/mL)	At time of diagnosis^1^	193.95±74.11	155.42±70.06	8.19±5.84	a-c, b-c
	7th day^2^	9.45±6.96	8.99±6.08		
IL-8 (pg/mL)	At time of diagnosis^1^	481.33±186.58	376.85±96.61	55.51±26.54	a-b, a-c, b-c
	7th day^2^	89.41±57.69	53.57±34.84		
TNF-α (pg/mL)	At time of diagnosis^1^	21.00±9.43	17.64±6.70	4.55±1.52	a-c, b-c
	7th day^2^	5.23±1.74	4.92±1.68		
**P value					
P<.05		1-2	1-2	—	—

* Kruskal Wallis test.

** Wilcoxon test.
